# Aging in Place and Healthcare Equity: How Rebuilding Together Supports Living Longer in Homes and Communities

**DOI:** 10.1093/geroni/igaf122.2819

**Published:** 2025-12-31

**Authors:** Christine McNichols, Annie Rhodes

**Affiliations:** Virginia Commonwealth University, Richmond, Virginia, United States; Virginia Commonwealth University, Virginia Commonwealth University, Virginia, United States

## Abstract

Aging in Place (AIP) refers to the ability to remain in one’s home and community as one ages. While AIP is widely regarded as beneficial, disparities in housing stability, accessibility, and affordability create inequitable barriers. Current clinical AIP interventions focus on individual-level solutions, often overlooking broader socio-economic and structural determinants. Using a Health Disparities Framework, demographic and service data from a community-based organization, Rebuilding Together Richmond (RT-R), was analyzed. Descriptive statistics assessed the characteristics of homeowners served, the types of repairs performed, and their potential impact on AIP. Repairs were categorized as structural or occupational to evaluate their contributions to home safety and accessibility. RT-R provided repairs for 33 homes, benefiting 47 individuals, all of whom were Black or African American living in a zip code with high eviction rates. The majority (63.8%) were female, and 51% were older adults and/or had a disability. Structural repairs were more frequent than occupational modifications, reflecting both homeowner needs, service availability, and community organizational goals. Housing stability is a critical yet overlooked factor in AIP. Integrating clinical AIP interventions with community-based solutions can more effectively address health disparities, reduce institutionalization risks, and improve long-term livability. Partnerships between healthcare practitioners and organizations like Rebuilding Together are essential to advancing equity in AIP. Access to housing is not accessible housing, and to remove barriers, practitioners and community-based organizations should expand their appreciation of obstacles to include historical, contemporary, economic, and environmental factors to work toward equity in aging in place for all.

